# Beyond actinic damage: Improvement of facial rhytides and lentigines with 5-aminolevulinate photodynamic therapy

**DOI:** 10.1016/j.jdcr.2025.08.040

**Published:** 2025-10-04

**Authors:** Milan Hirpara, Sarah Choe, Luke Horton, Joel Cohen, Natasha Mesinkovska

**Affiliations:** a Department of Dermatology, University of California, Irvine, Irvine, California; b AboutSkin Dermatology and AboutSkin Research, Greenwood Village, Colorado

**Keywords:** 5-aminolevulinate, actinic keratosis, laser resurfacing, lentigines, photoaging, photodynamic therapy, pigmentation, rhytides

## Introduction

Actinic keratosis (AK) is the third most diagnosed skin condition among all medical specialties and the most common visit diagnosis among dermatologists.^1^ Field-directed therapies offer an approach to treat multiple AKs simultaneously, but the potential side effects compromise tolerability and discourage patients from opting for these treatments. Providing evidence of additional photoaging benefits may stimulate greater patient interest, leading to enhanced satisfaction and adherence.

Although 5-fluorouracil cream has demonstrated greater efficacy in AK clearance, photodynamic therapy (PDT) has gained interest for its combined effects in treating AKs and photorejuvenation.^2^^,^^3^ Several reports investigated PDT’s ability to improve photoaging-related features such as facial rhytides and lentigines, with limited findings due to lack of validated assessment scales, facial subunit-specific grading, and consistent protocols, leaving the measurable benefits unclear.^4^, ^5^, ^6^

Although PDT is not considered an anti-aging procedure and is primarily used for the treatment of field cancerization, its noninvasive nature and combined medical and skin-enhancing benefits make it a compelling option for patients requiring treatment for AKs. This study aims to better evaluate the effects of 5-aminolevulinate (5-ALA) PDT in improving the appearance of facial rhytides and lentigines, by using currently validated facial subunit scales to quantify the efficacy of PDT for further insight into its photoaging benefits.

## Methods

### Study population

Adult patients (≥18 years of age) who underwent 5-ALA PDT for the treatment of facial AKs were retrospectively identified using our institutional neoplastic skin conditions registry from August 2024 to February 2025. Patients were included if they had undergone PDT for the treatment of facial AKs. At our institution, PDT is delivered using a standardized protocol consisting of a 90-minute incubation with 17% 5-ALA, followed by blue light illumination (400 nm) for 16 minutes and 40 seconds. Eligibility required the availability of standardized baseline and follow-up facial images (12-20 weeks) obtained using a three-dimensional (3D) imaging system (Vectra XT, Canfield Scientific). For patients who underwent multiple PDT sessions, only data from the first treatment were included in the analysis. Patients were excluded if they had received any botulinum toxin or dermal filler treatments within 1 year before PDT, or at any time between completion of PDT and the follow-up visit.

### Data collection and outcome assessment

Demographic and clinical data were extracted from medical records, including age, sex, race, ethnicity, skin type, and time from treatment to follow-up.

Outcomes were assessed by 3 independent dermatologists reviewing standardized pre- and post-treatment 3D images. The following validated photonumeric scales were used to assess photoaging features: separate 5-point scales for facial rhytides (Merz Scale, Merz Pharmaceuticals) in the following areas: forehead, glabella, periorbital, melomental, nasolabial, and perioral regions.^7^ Superficial lines on the cheeks were also evaluated using a 5-point scale for fine lines over the midface (Allergan Fine Lines Scale, AbbVie).^8^ Solar lentigines were assessed using a separate 5-point photonumeric scale, and overall photoaging improvement was measured using the 7-point Global Aesthetic Improvement Scale (GAIS).^9^

### Statistical analysis

Descriptive statistics were used to summarize patient demographics, follow-up duration, and treatment outcomes. Changes in photonumeric scores for facial rhytides and solar lentigines were calculated as the difference between baseline and post-treatment values. GAIS scores, which reflect an overall comparative assessment between baseline and follow-up images, were reported descriptively.

## Results

### Demographics

A total of 15 patients (9 males and 6 females) with a mean age of 65.9 ± 11.3 years were included in the analysis. All patients self-identified as White and non-Hispanic. The patients were Fitzpatrick skin type I (26.7%), type II (60.0%), and type III (13.3%). The mean time at follow-up imaging was 4.0 ± 1.8 months after treatment.

### Outcomes

**Fig 1:**
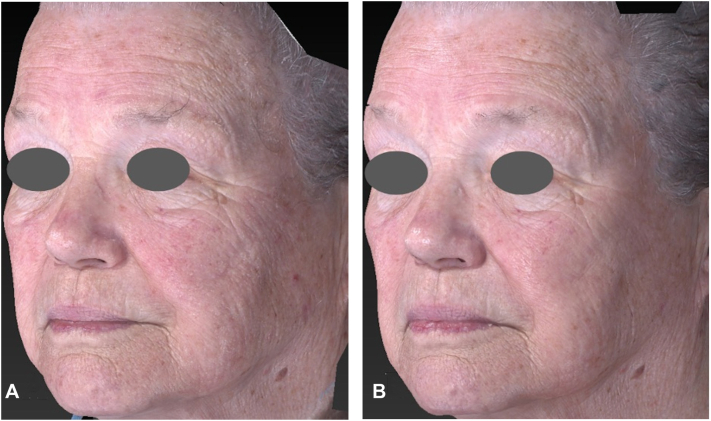
Three-dimensional imaging of an 82-year-old female before **(A)** and 12 weeks after **(B)** 5-aminolevulinate photodynamic therapy to face. Quantitative changes in rhytid severity were observed in the periorbital area (−1 ± 0), left cheek (−1 ± 0), and right cheek (−1.33 ± 0.58) with no change in melomental folds (0 ± 1). Lentigines severity decreased by −1.33 ± 0.58 and GAIS score was 1.33 ± 0.58. *GAIS*, Global Aesthetic Improvement Scale.

**Fig 2:**
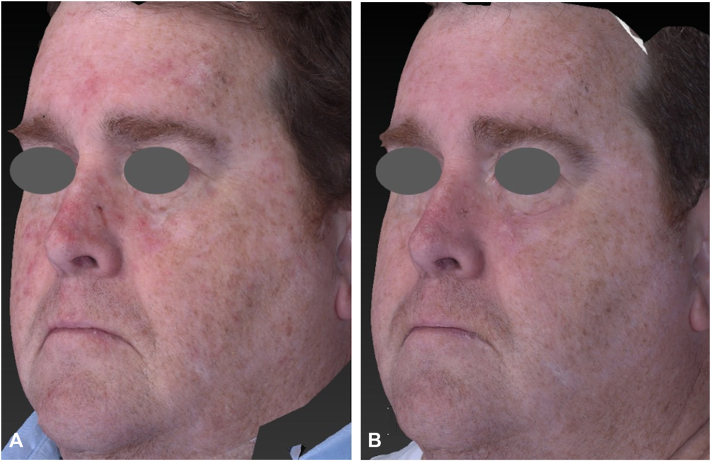
Three-dimensional imaging of a 55-year-old male before **(A)** and 20 weeks after **(B)** 5-aminolevulinate photodynamic therapy to face. Lentigines severity decreased by −1 ± 0 and GAIS score was 1.67 ± 0.58. Baseline rhytides scores in this patient were low. Quantitative changes in rhytid severity observed in periorbital area (−0.67 ± 0.58), melomental folds (−0.67 ± 0.58), and perioral region (−0.33 ± 0.58), with no change in the left or right cheek (0 ± 0). *GAIS*, Global Aesthetic Improvement Scale.

The mean GAIS score across all 3 reviewers was 1.2 ± 0.48, corresponding to a rating between “improved” and “much improved” (Table I). The average change in rhytid scores across all 3 evaluators showed consistent improvement. Mean change in scores across all 3 reviewers showed the following improvements: forehead (−0.49 ± 0.59), glabella (−0.53 ± 0.55), periorbital (−0.64 ± 0.53), nasolabial (−0.40 ± 0.50), melomental (−0.53 ± 0.59), and perioral (−0.60 ± 0.50) (Fig 1). Mean score reductions for midface superficial lines were −0.49 ± 0.55 for the left cheek and −0.58 ± 0.54 for the right cheek. Lentigines severity demonstrated a mean score reduction of −1.0 ± 0.35 across all patients (Fig 2).

**Table I:** Patient characteristics and change in rhytides, lentigines, and Global Aesthetic Improvement Score

	Patients (*n* = 15)
Age (mean ± SD; y)	65.9 ± 11.3
Age range	43-83
Female (%)	6 (40%)
Race and ethnicity (%)	
Caucasian	15 (100%)
Hispanic	0 (0%)
Follow-up time (mean ± SD; mo)	4.0 ± 1.8
Change in rhytid severity (mean ± SD)	
Forehead	−0.49 ± 0.59
Glabella	−0.53 ± 0.55
Periorbital	−0.64 ± 0.53
Nasolabial	−0.40 ± 0.50
Melomental	−0.53 ± 0.59
Perioral	−0.60 ± 0.50
Left cheek	−0.49 ± 0.55
Right cheek	−0.58 ± 0.54
Change in lentigines (mean ± SD)	−1.0 ± 0.35
GAIS (mean ± SD)	1.2 ± 0.48

*GAIS*, Global Aesthetic Improvement Score; *SD*, standard deviation.

Females experienced greater reductions in rhytid scores than males in the nasolabial folds (−0.56 ± 0.51 vs −0.30 ± 0.47) and in both left (−0.67 ± 0.59 vs −0.37 ± 0.49) and right cheeks (−0.72 ± 0.57 vs −0.48 ± 0.51). Severity of lentigines also decreased more in females (−1.06 ± 0.42) than in males (−0.96 ± 0.44). The mean GAIS score was 1.06 ± 0.54 for females and 1.30 ± 0.67 for males.

## Discussion

This study demonstrates the measurable photoaging improvements in facial subunit analysis in both rhytid severity and solar lentigines with 5-ALA blue light PDT in patients undergoing treatment for facial AKs. Across all evaluated facial regions, patients experienced consistent reductions in rhytid scores, with the most pronounced improvements observed in the periorbital (−0.64 ± 0.53), perioral (−0.60 ± 0.50), cheeks (−0.49 to 0.58 ± 0.55), and glabella (−0.53 ± 0.55) regions. The results observed here augment findings from previous reports evaluating PDT photoaging benefits, by providing detailed facial-subunit analysis, scores on validated scales and reproducible, consistent protocols.^4^, ^5^, ^6^

The strongest component of this data analysis is that the scales used provide comparison with other available procedures for cosmetic benefits, such as laser and light devices. When compared to results reported for rhytidectomy with CO_2_ fractional laser resurfacing at 3 months, the values are surprisingly close: laser −0.67 in the infraorbital hollows (vs −0.64 with PDT), −0.33 in the nasolabial folds (vs −0.40 with PDT), −0.67 in the marionette lines (vs −0.53 with PDT), and −0.50 in the lips (vs −0.60 with PDT). In that study at 12 months, improvements increased to −1.0, −0.67, −1.0, and −0.75 points in the same regions, respectively.^10^ The results from this PDT study at a mean follow-up of approximately 4 months were comparable to those at 3-month timepoint in the CO_2_ laser resurfacing cohort, suggesting non-inferiority in the short term. It will be interesting to follow our cohort, as we expand the data and see if the same observations hold at longer timeframes. Similarly, the GAIS improvements seen in our study were comparable to reports with established energy-based photoaging treatments, such as radiofrequency.^11^

The mechanism of photorejuvenation with PDT involves the production of reactive oxygen species which ultimately induces collagen remodeling.^12^ Upon topical application, 5-ALA is converted into protoporphyrin IX, a photosensitizer that preferentially accumulates in epidermal cells where it generates reactive oxygen species, leading to controlled oxidative damage, apoptosis, and exfoliation of photodamaged keratinocytes. This acute injury initiates a localized inflammatory cascade, stimulating dermal fibroblast activity and subsequent neocollagenesis.^13^^,^^14^ These secondary effects may explain the observed improvement in skin texture and reduced fine lines.

Recent single-cell RNA sequencing studies on human skin demonstrated that photoaged skin is characterized by increased cellular senescence, reduced T cell function, and diminished antigen presentation by dendritic cells.^15^ Treatment with ALA-PDT reversed many of these changes, improving immune cell activity, restoring dendritic cell function, and enhancing cell-to-cell communication in the skin immune microenvironment.^15^ These findings suggest that ALA-PDT not only improves photoaging clinically, but also rejuvenates the local immune landscape, offering a promising strategy for reversing skin and possibly systemic aging.

A notable reduction in severity of solar lentigines (−1.0 ± 0.35) was observed following PDT treatment, which was unexpected. Typically, PDT is not considered effective for removing lentigines, as these lesions are not characterized by rapid cell turnover. Review of potential mechanisms suggests that PDT may downregulate melanogenesis by directly suppressing tyrosinase activity in melanocytes and by reducing the secretion of melanocyte-stimulating cytokines, such as KIT ligand and hepatocyte growth factor from keratinocytes and fibroblasts.^12^

Although the primary indication for PDT remains the treatment of AKs, its recognized photorejuvenation benefits may alter future insurance coverage policies. If patients begin to pursue PDT primarily for its cosmetic benefits, insurance providers may be inclined to use a standardized AK severity scale to determine eligibility for coverage. However, no such universally adopted, standardized AK severity scale currently exists.

This study adds to the growing evidence that PDT offers significant benefits for photoaging, as demonstrated in a small, controlled patient cohort. The primary study limitations include the homogeneity of patient age, race, and skin type—characteristics that mirror the typical population receiving this therapy but restrict the generalizability of our findings to more diverse groups. Observer bias may have influenced outcome scoring, as the reviewing physicians were not blinded to the pre- and post-treatment image sets. Additionally, the male predominance in our cohort was associated with generally lower baseline wrinkle severity with limited room for observable improvement. This could have potentially underestimated the full photorejuvenation potential of PDT in women and populations with more pronounced photoaging, which we are addressing by expanding the cohort.

Future studies incorporating randomized controlled trial designs, larger and more diverse patient populations, and comparisons to other therapeutic approaches will help validate and extend these findings. Comparative studies also evaluating variations in treatment protocols, such as different photosensitizer formulations, concentration, or incubation duration, as well as alternative light sources, could provide valuable insights into optimizing PDT for both therapeutic and photoaging outcomes. Additionally, developing a standardized AK severity grading system may allow for assessment of whether photorejuvenation outcomes correlate with baseline AK severity and post-treatment inflammatory response.

## Conclusion

PDT with 5-ALA was shown to effectively improve facial rhytides, lentigines, and overall skin appearance in this small cohort of subjects treated for actinic keratoses. These findings reinforce PDT's role as a versatile option for addressing field cancerization while providing measurable photorejuvenation benefits. Given its favorable cost profile and coverage by most insurance plans, PDT offers both clinical and financial practicality for patients and providers. Educating patients on these potential benefits may improve treatment adherence and overall satisfaction.

## Conflicts of interest

None disclosed.
